# Situs inversus totalis with congenitally corrected transposition of the great arteries: insights from cardiac MRI

**DOI:** 10.1186/2193-1801-3-601

**Published:** 2014-10-15

**Authors:** Jan M Sohns, Michael Steinmetz, Heike Schneider, Martin Fasshauer, Wieland Staab, Johannes Tammo Kowallick, Andreas Schuster, Christian Ritter, Joachim Lotz, Christina Unterberg-Buchwald

**Affiliations:** Institute for Diagnostic and Interventional Radiology, Georg-August-University Göttingen, UMG Universitätsmedizin Göttingen, Robert-Koch-Str. 40, 37099 Göttingen, Germany; DZHK (German Centre for Cardiovascular Research), partner site Göttingen, Göttingen, Germany; Department of Pediatric Cardiology and Intensive Care Medicine, Georg-August-University, Göttingen, Germany; Department of Cardiology and Pneumology, Georg-August-University Göttingen, Göttingen, Germany

**Keywords:** Situs inversus, Cardiac MRI, Cardiovascular imaging, Transposition of the great arteries, CMRI, Congential heart disease

## Abstract

**Introduction:**

Situs inversus totalis with congenitally corrected transposition of the great arteries represents a relatively rare congenital condition.

**Case description:**

The current report describes the case of a 56 year old patient with an atrio-ventricular and ventricular-arterial discordance of the heart chambers without surgical correction, incidentally detected during hepatocellular carcinoma evaluation. The systemic venous blood arrived via the right atrium and a mitral valve in the morphologically left but pulmonary arterial ventricle that gave rise to a pulmonary trunk. The pulmonary venous blood passed the left atrium and the tricuspid valve into a morphologically right but systemic ventricle that gave rise to the aorta.

**Discussion and evaluation:**

The switched anatomy was incidentally detected on echocardiography. The patient was referred to cardiac magnetic resonance imaging (CMR) including flow measurements, volumetry and late enhancement. CMR results showed a mildly impaired function and the switched anatomy. During a follow-up period of 2 years the patient was suffering from only mild heart failure and dyspnea.

**Conclusions:**

Heart failure symptoms and arrhythmias can appear with increasing age in patients with congenitally corrected transposition. Early CMR allows accurate diagnosis and timely introduction of adequate therapy thereby avoiding disease progression.

## Background

Total situs inversus is a rare diagnosis (Roongruangchai et al. 2012; Iino et al. 2012; Garg et al. 2003; Schmidt et al. 2000; Hoeffel 2001). It can be associated with transposition of the great thoracic arteries (TGA), either of the D- or L-type (i.e. congenitally corrected) TGA (Hoeffel 2001; Warnes 2006; Graham et al. 2000; Patanè et al. 2008; Dyer & Graham 2003; Shin’oka et al. 2007). The variation of pressure and cardiac workload can damage the myocardium in switched ventricels (Patanè et al. 2008; Dyer & Graham 2003). Affected patients may develop cardiomyopathy with reduced systolic ejection and arrhythmia if they are not subjected to adequate therapy or early surgery (Hoeffel 2001; Shin’oka et al. 2007). Cardiovascular resonance imaging (CMR) is a powerful non-invasive imaging tool for the characterization of cardiac anatomy in total situs inversus (Schmidt et al. 2000).

## Case description

**Figure 1:**
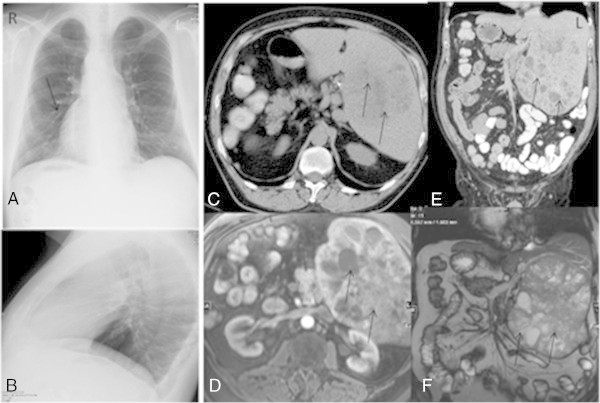
**Imaging of situs inversus before CMRI. A**: Chest X-ray with dextro-cardia (black arrow), posterior anterior projection, initial image of the first appearance in our hospital in January 2010. **B**: Lateral view of the chest. **C**: CT in axial and coronal **(E)** view after intra-venous contrast medium application showing a situs inversus totalis of the abdomen. The liver is on the left side of this axial view showing a hepatocellular carcinoma infiltrating a liver lobe (black arrows; 64-VCT Light Speed, GE, Healthcare, USA). The same view was seen in transversal **(D)** and coronal **(F)** views after application of contrast medium in 1.5 T MRI (Magnetom Symphony, Siemens AG, Healthcare sector, Erlangen, Germany). **C**: Coronal view in CT and MRI with contrast medium. The spleen was right sided (not demonstrated on these slices).

A 56-year old patient was referred to our center with hepatocellular carcinoma for further diagnostic work-up. Incidentally, an inversion of the liver and abdominal structures to the opposite side was seen on computed-tomography (CT) and abdominal magnetic-resonance imaging (MRI). During preoperative clinical check-up and postoperative follow-up after partial liver resection (Figure 1), standardized cardiac imaging including Chest X-ray and echocardiography were performed. Pre-operative diagnostics demonstrated suspected pulmonary artery hypertension, elevated flow of the atrio-ventricular valve and higher pressure in the supposed right ventricle. Consequently, complete cardiac work-up was performed in the adult congenital heart disease unit.

General clinical examination documented the following findings: The patient suffered from vertigo after getting up fastly and dyspnea during physical exercise. His skin color was rather pale. There was no edema of his legs, and no nycturia was reported. Blood pressure of the right arm was about 130/65 mmHg, and 140/65 mmHg of the right leg. There was a heart rate around 90 heartbeats (hb) per minute. Nutritional and general conditions were normal. Transcutaneous oxygen saturation was about 100% and breathing was normal with eupnoea. There were no suspicious breathing sounds of the lungs. The peripheral pulse status was normal.

**Figure 2:**
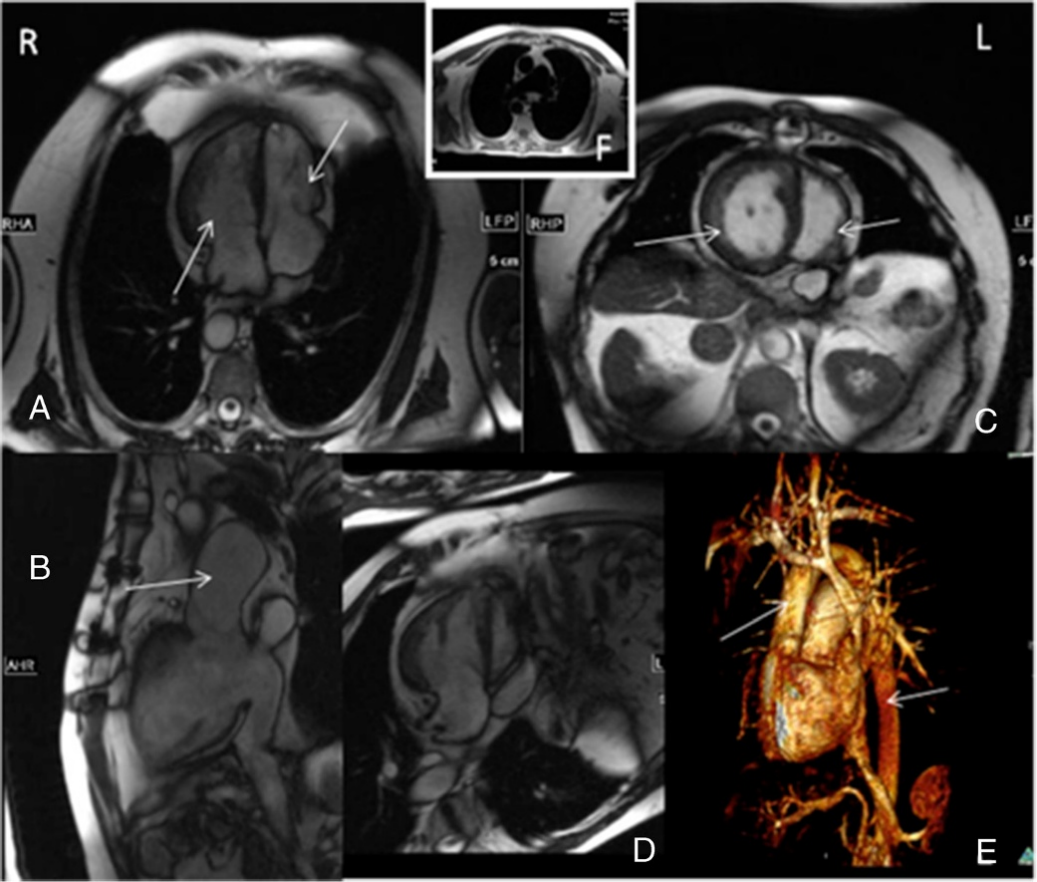
**Situs inversus in CMRI.** 1.5 T MRI (Magnetom Symphony, Siemens, Healthcare sector, Erlangen, Germany) after application of contrast medium. **A**: Four-chamber-view with a morphologic right and systemic ventricle (with hypertrophic trabecular structures) as well as a small smoother sub-pulmonary ventricle on the left side (white arrows). **B**: A dilated pulmonary artery (white arrow) is detected **(A)**. **C**: Short axis or two-chamber-view (white arrows) demonstrating the cardiac ventricular anatomy of the two ventricles. **A** D**-**shaped septum bulging from the systemic right ventricle towards the morphological left ventricle connecting to the pulmonary circulation can be appreciated. **D**: The outflow of the aortic arch is shown in this image, coming from the functional left ventricle, morphological (original) right ventricle. **E:** The three-dimensional reconstruction shows the left-sided aorta and right-sided pulmonary trunk (white arrows). **F**: This image demonstrates the dilated pulmonary trunk and proximal pulmonary arteries in the initial Haste-sequences.

The electrocardiogram (ECG) at rest showed a normal sinus rhythm with precordial ST- segment depression (V2 to V4), T-wave inversion (V2 to V3) and signs of biventricular hypertrophy.Echocardiography (Echo) showed a situs inversus totalis with meso- or dextrocardia of the heart. There was a side-by-side position of the great thoracic vessels with suspected atrio-ventricular discordance and ventricular-arterial discordance. We detected an L-transposition or congenitally corrected transposition of the great thoracic vessels. The ventricle of the right side (Figure 2) was hypertrophic with more trabecular structures and a typical right ventricular morphology. This ventricle appeared with impaired function. On Echo it was not possible to assess the ejection of this ventricle in detail. There was mild regurgitation of the atrio-ventricular (AV) valve. This AV-valve was located more apically and right-sided.

The ventricle on the left side of the septum appeared as a morphological original left ventricle with less trabecular structures and relatively smooth myocardial wall structures. The AV-valve was located more to the base of the heart and the valve appeared like a morphological mitral valve. The outflow vessel of this ventricle appeared as a pulmonary artery or pulmonary trunk with a low-grade stenosis (peak velocity 1.6 m/sec). There was low-grade AV-valve regurgitation (functional tricuspid valve) and a relatively high right ventricular pressure of 30 mmHg. The aortic arch ascended steeply dorsal and was located rather right and medial during descent.

A detailed anatomical view was demonstrated on CMR using ECG-triggered HASTE and True-FISP-sequences and a segmental approach. The following features were found: The pulmonary veins drained into a morphologically left atrium. The atrio-ventricular valve connecting the left atrium to the ventricle was tricuspid-shaped and located more to the apex, with moderate regurgitation. A hypertrophic ventricle with increased trabeculation, reduced ejection fraction, a moderator band and an infundibulum below the arising great vessel was identified as a morphologically right but functionally systemic ventricle. It gave rise to an anteriorly positioned aorta on the right side, with steeply ascending aortic arch to the dorsal side. The blood from the right upper body side flowed relatively normal via a transversal vein into the left-sided superior vena cava. There was no persisting right superior vena cava. Following the caval veins, the blood drained into a morphologically right atrium, which was connected to a mitral-shaped atrio-ventricular valve. The valve was located more to the base of the heart. The lateral and medial leaflets of the valve were asymmetric with pointed leaflets of the medial valve parts. A smoothly muscled, compact ventricle was connected to this valve, identified as a morphologically left, functionally pulmonary-arterial ventricle. The pulmonary artery was located on the left side related to the aorta and preceded dorsal. The pulmonary arteries were dilated, which can also be seen in patients with pulmonary hypertension or after pulmonary artery embolism. Pulmonary hypertension can be a limiting factor in these patients with increasing heart failure (Roongruangchai et al. 2012; Shin’oka et al. 2007).

The morphologic right, systemic ventricle had an end-diastolic volume of 179 ml (normal: 101–236 ml) and an ejection fraction of 47% (normal: 55–74%).

The morphologic left, sub-pulmonary ventricle had an end-diastolic volume of 83 ml (normal: 110–243 ml), an ejection fraction of 53% (normal: 47–63%).

## Discussion and evaluation

The patient had a congenital heart defect with situs inversus totalis and atrio-ventricular and ventricular-arterial discordance.

L-transposition of the great thoracic vessels is very rare (Roongruangchai et al. 2012; Iino et al. 2012; Garg et al. 2003; Schmidt et al. 2000; Hoeffel 2001). This congenital cardiac defect remains often asymptomatic until adulthood (Hoeffel 2001; Warnes 2006; Graham et al. 2000; Patanè et al. 2008; Dyer & Graham 2003; Shin’oka et al. 2007). When patients are older, heart failure and cardiac arrhythmias can appear. This is caused by progressing failure of the morphological right systemic ventricle that is burdened with high systemic pressures (Schmidt et al. 2000; Shin’oka et al. 2007). A mildly reduced function was already detected in this patient of the right-sided systemic ventricle.

After a pulmonary embolism in September 2010 the patient received Phenprocoumon (INR-adapted 2–2.5). The patient attends frequent outpatient clinics in the unit for adults with congenital heart defects in our hospital with regular CMR scanning. After an interval of two years, in November 2012, after the initial CMR in January 2010, the global function of the heart was stable on CMR and Echo. Whilst the patient had complained about increasing dyspnea at rest, the actual ejection fraction (11/2012) was however stable with 45% (47% in 1/2010) of the systemic ventricle (morphologic right) and 62% (53% in 1/2010) of the morphologic left (pulmonary arterial ventricle).

It is important to consider the presence of such defects in the adult population. This is underpinned by the fact that the patient had undergone an invasive closure of an atrial-septal defect in 1972 (secundum type). At this time, the situs inversus and the unusual heart anatomy were – surprisingly - not detected and no specific diagnostics followed.

## Conclusion

This case describes the changes in a mid-aged patient with an atrio-ventricular and ventricular-arterial discordance of the heart chambers (L-Transposition of the Great Arteries or congenitally corrected TGA (ccTGA)) without surgical correction. Early CMR allows accurate diagnosis and monitoring of disease progression. The information that can be derived from CMR is useful for timely introduction of adequate therapy allowing optimal treatment with improved quality of life in patients with incidentally detected congenital heart defects.

### Patient’s consent

Written informed consent was obtained from the patient for publication of this case report and any accompanying images. A copy of the written informed consent is available for review by the Editor of this journal.

## Abbreviations

AV: Atrio-ventricular
CMRI: Cardiac resonance imaging
CT: Computed tomography
ECG: Electrocardiography
Echo: Echocardiography
HASTE: Half Fourier Acquisition Single Shot Turbo Spin Echo
MRI: Magnetic resonance imaging
TGA: Transposition of the Great Arteries
True-FISP: True fast imaging with steady state precession.
